# Evaluation of the Serological Point-of-Care Testing of Infectious Mononucleosis by Data of External Quality Control Samples

**DOI:** 10.1177/1178636120977481

**Published:** 2020-12-03

**Authors:** Salla J Kiiskinen, Oskari Luomala, Teija Häkkinen, Susanna Lukinmaa-Åberg, Anja Siitonen

**Affiliations:** 1 Expert Microbiology Unit, Finnish Institute for Health and Welfare (THL), Helsinki, Finland; 2 Infectious Disease Control and Vaccinations Unit, Finnish Institute for Health and Welfare (THL), Helsinki, Finland; 3 Labquality Ltd, Helsinki, Finland; 4 Fimlab Laboratoriot Oy, Lahti, Finland

**Keywords:** external quality assessment, external quality control, proficiency testing, clinical laboratory, serological point-of-care test, Epstein-Barr virus, infectious mononucleosis

## Abstract

Timely and reliable laboratory diagnostics is a necessity for patient safety and good patient management. Success in external quality assessment (EQA) reflects on the everyday work in a clinical laboratory. This study evaluated the reliability of serological point-of-care (POC) testing for the Epstein-Barr virus (EBV) that causes infectious mononucleosis (IM). Data from the results of 95 external quality control (EQC) samples, altogether 18 885 results during an eight-year period (2010-2017) were collected from 273 Finnish testing sites. Diagnosing acute infectious mononucleosis (EBV IM) is based on clinical, haematological and serological findings. Heterophile antibody tests are used for this purpose because they can be carried out at POC and are cheap and robust to perform. In this study, the data showed that the testing sites used 3 test methods and 17 different test kits; of the kits, 4 were used during the whole study period. The most commonly used test methods were immunochromatographic assays (12 test kits, 17 959 EQC results). Latex agglutination (4 test kits, 504 results) and immunofiltration test methods (one kit, 422 results) were also used. The overall success rate was 99.3% (for positive samples 99.6%, for negative samples 99.1%). The success rates of the different test methods varied from 94.3% for the immunofiltration method to 99.6% for the latex agglutination method. The lowest success rates were found for negative samples: 82.0% (QuickVue, Quidel [immunochromatographic method]), 91.3% (RDT EBV IgM Assay, Bio-Rad [immunofiltration method]). The results of the negative samples that represented old EBV immunity were the most difficult to interpret with a success rate of 98.9% compared to success rates of clearly positive (99.6%) and negative (99.5%) samples (*P* < .001). Especially the immunofiltration method (RDT EBV IgM Assay) produced 13.7% false positive results for samples of old immunity. The data showed that 42 of the studied 95 EBV IM EQA rounds were reported as expected (true positive or true negative) by all testing sites.

## Introduction

The purpose of laboratory methods in clinical microbiology is to detect causative agents of infections. Tests conducted on patients outside the traditional clinical laboratory setting are called point-of-care (POC) tests. POC tests are rapid tests that are relatively easy to use, inexpensive and usually shorten the turnaround time and, thus, speed up patient management. While clinical laboratories have become more centralised, POC testing in other testing sites has become increasingly common.^1^ At best, POC test results can have an immediate effect on patient care.

The Epstein-Barr virus (EBV) is found all over the world and most people get infected with it at some point of their lives.^2,3^ EBV infection usually occurs during infancy or childhood. The infection can be asymptomatic but EBV can also cause infectious mononucleosis (IM) with symptoms including sore throat, fever and fatigue. EBV IM, also called glandular fever, is most common among teens and young adults.

Diagnosing EBV IM can be challenging because the symptoms may resemble, for example, some malignant haematological diseases. Therefore, a false positive result may have serious clinical consequences due to the delay in initiating the right treatment. The diagnosis of EBV IM is based on clinical, haematological and serological findings.^4^ Heterophile antibody tests are commonly used for this purpose because they are cheap to perform and robust. If needed, the infection can be confirmed with a blood test that detects EBV-specific antibodies. Serological POC tests, including EBV IM test kits and test methods, have improved during the past decades^5-8^ and at this moment a selection of POC tests are commercially available for EBV IM. Even though they are simple to use in general, it is important to know the limitations of the test kit which has been selected for use.

The aim of this study was to evaluate the following: (1) are the results of the POC tests for EBV IM reliable and consistent regardless of the site of testing, (2) how many tests and what kind of the methods are in use and (3) do these tests and methods differ in their performance. For this purpose, we collected the data of the results of the external quality control (EQC) EBV IM samples of EQA rounds from an eight-year period 2010 to 2017 in Finland. In all, data from 18 885 EBV IM POC results of 273 testing sites were analysed.

In Finland all investigations of communicable diseases, including POC tests, can only be done in testing sites that are approved for this purpose by the Regional State Administrative Agencies (RAAs).^9^ The detailed procedures for implementing the legal regulations, called the licensing system for clinical microbiology laboratories, were created in 1993.^10^ The aim of this mandatory licensure is to assure comparable and reliable performance in all testing sites investigating clinical microbiology specimens. The Finnish Institute for Health and Welfare (THL) maintains a register of all these testing sites and the microbiological specimens they investigate.

In order to be approved, a testing site has to have the appropriate premises and equipment, and competent staff for performing its tasks. Also, one of the basic requirements is obligatory participation in external quality assurance (EQA) rounds at least 4 times per test item per year. According to the communicable diseases act, the THL and RAAs give free access to the data of all the EQA results of every testing site. Most of the testing sites carrying out microbial POC tests using commercial test kits are small health care centres.

## Materials and Methods

### Data collection

The testing sites that participated in EBV IM EQA rounds were different-sized laboratories and health centres (called testing sites in this study) from both the public and private sector. The data was grouped according to the size of the testing sites: small testing sites (n = 155) examined only 50 or less (median 45) IM patient samples per year and large testing sites (n = 118) examined 51 or more (median 120) samples per year. This information was available in the THL’s register. Altogether, data on results of 273 testing sites was collected.

According to the THL’s register the most commonly used provider of the EQA rounds for EBV IM POC is Labquality Ltd. It is an independent Finnish service company focusing on the quality assurance of medical laboratories and POC testing sites. At Labquality Ltd, EBV IM POC EQA rounds have been available since 1987. The scheme is accredited according to the ISO 17043 standard.^11^ During the 8 years (2010–2017), in 32 EQA rounds, altogether 96 EQC samples had been sent by Labquality to the 273 testing sites.

All the EQC samples were liquid human plasma, and each batch originated from a single human donor. The same plasma batch was used 1–3 times if the original volume was sufficient. Before distribution, each EQC sample had been pretested by an expert Finnish clinical microbiology laboratory. This pretesting consisted of the investigations of heterophile antibodies, EBV-IgM, the EBV nuclear antigen EBNA-IgG, EBV-IgG antibodies and the avidity of IgG antibodies. The interpretation of results in EBV IM POC is qualitative (positive or negative).

The 32 rounds consisted of 36 positive and 60 negative EQC samples from 74 different plasma batches. The data of one negative EQC sample was excluded from this study because lack of consensus in reported results. This left data on 95 samples with 18 885 results for analyses.

For further analyses, the data of the EQC results was divided into 3 groups according to the clinical interpretation. In Group I (n = 36), the samples were positive for heterophile antibodies EBV-IgG and EBV-IgM, negative for EBNA-IgG and low in IgG avidity, and they were graded as *Positive, recent EBV infection*. In Group II (n = 23), samples were negative and graded as *Negative, no EBV antibodies*. In Group III (n = 36), the samples were negative for heterophile antibodies and EBV-IgM, positive for EBV-IgG and EBNA-IgG, and high in IgG avidity, and they were graded as *Negative, old EBV immunity*.

### Statistical analyses

The Fisher exact test and the chi-square test were used to compare the results between large and small laboratories, test methods and specimen groups. For the more complex associations, logistic regression analysis was used.^12^ A *P*-value <.05 was considered to indicate statistical significance.

## Results

In this study, data on 18 885 EBV IM EQC results reported by 273 testing sites using altogether 3 test methods and 17 different test kits were available for analyses (Table 1). Of all the results analysed, 99.3% were correct. Latex agglutination, immunochromatographic and immunofiltration tests methods gave 99.6%, 99.4% and 94.3% correct results, respectively. The most commonly used tests methods were immunochromatographic methods (12 test kits, 17 959 EQC results). Some latex agglutination methods (4 test kits, 504 EQC results) and one immunofiltration method (1 test kit, 422 EQC results) were also used.

**Table 1.** The external quality control results, test methods and used test kits during 2010–2017.

Test methodTest kit (manufacturer)	Expected result	Reported results: true/all	Success %
Latex agglutination tests for heterophile antibodies		502/504	99.6
Monogen^a^ (Biokit)	pos.	132/133	99.2
	neg.	177/178	99.4
Monospot^a,b^ (Meridian)	pos.	64/64	100
	neg.	102/102	100
Latex agglutination, other^c^	pos.	12/12	100
	neg.	15/15	100
Immunochromatographic assays for heterophile antibodies		17 855/17 959	99.4
Clearview^a,d^ (Unipath/Alere)	pos.	4609/4618	99.8
	neg.	7099/7148	99.3
InstAlert^a,e^ (Innovacon)	pos.	1800/1811	99.4
	neg.	2964/2981	99.4
Mononucleosis^d,f^ (SureScreen Diagnostics)	pos.	235/238	98.7
	neg.	454/456	99.6
Mnitop (All. Diag)	pos.	87/87	100
	neg.	162/162	100
Nadal Mononucleosis^f^ (Nal von Minden)	pos.	36/38	97.7
	neg.	99/100	99.0
OSOM Mono Test (Sekisui Diagnostics)	pos.	28/28	100
	neg.	67/67	100
QuickVue (Quidel)	pos.	26/26	100
	neg.	41/50	82.0
Immunochromatographic, other^g^	pos.	57/57	100
	neg.	91/92	98.9
Immunofiltration test for IgM antibodies		398/422	94.3
RDT EBV IgM Assay^h^ (Bio-Rad)	pos.	146/146	100
	neg.	252/276	91.3
All test methods		18 755/18 885	99.3
17 test kits	pos.	7232/7258	99.6
	neg.	11 523/11 627	99.1

a The test kit was in use for the whole eight-year study period.

b The test kit was only used in large laboratories.

c Contains the results from 2 tests: Avitex-IM (Omega Diagnostics) and MNI Test (Fumouze).

d Contains one possible sample mix-up.

e Contains two possible sample mix-ups.

f The test kit was only used in small laboratories.

g Contains the results from 5 tests: MNITOP optima im (Biosynex), Diaquick Mononucleosis Cassette (Dialab), Mono Rapid Test Cassette (Hangzhou Alltest biotech), Immunocard Stat Mono (Meridian) and Mononucleosis Test Card (ulti med).

h The test kit only had manufacturer validation for serum samples.

The number of testing sites per year varied during the eight-year study period as some testing sites finished their microbiological operations and new ones started (data not shown). Of the 17 test kits, 4 were used during the whole examination period (Table 1). The data of the results of 2 latex agglutination and 5 immunochromatographic test kits were grouped together due to too few results during the whole study period.

Altogether, 130 false results were given. Of these, 74 negative samples were reported incorrectly as positive and 26 positive samples incorrectly as negative. Clearview produced 58 and InstAlert 28 false results, both most often for negative samples. The RDT EBV IgM assay that was validated for serum samples led to false positive results for 24 plasma samples (Table 1). The QuickVue test kit had the worst performance for negative samples (correct results in 82%).

The test kits in use differed between the different sizes of testing sites. Monospot, the MNI Test and Mnitop were only used in large testing sites with 51 or more EBV IM patient samples per year. Mononucleosis and Nadal Mononucleosis were only used by small testing sites with 50 or less EBV IM patient samples per year. Monogen, Clearview, InstAlert, OSOM Mono Test, QuickVue and the RDT EBV IgM Assay were used in both sizes of testing sites. Of the studied 95 EQC rounds, 42 were repeatedly and expectedly reported as either true positive or true negative by all of the participating testing sites. Of the 130 false EQC results, 46 were from EQA rounds that yielded one or two reported false results per round. No testing site stood out with multiple false results (data not shown).

The grouping of the data in 3 groups showed differences between EQC samples representing different clinical interpretation (Table 2). In Group I (*Positive, recent EBV infection*), the latex agglutination, immunochromatographic and immunofiltration test methods gave 99.5%, 99.6% and 100% correct results, respectively. In Group II (*Negative, no EBV antibodies*), the same methods gave 99.1%, 99.5% and 98.3% correct results, respectively, and similarly in Group III, EQC (*Negative, old EBV immunity*), the success percentages were 100%, 99.1% and 86.4% respectively. The EQC samples in Group III produced statistically significantly more wrong results than the samples in Groups I and II (correct percentages 98.9% vs 99.6% and 99.5%; *P* < .001; Table 2).

**Table 2.** External quality control results according to the used test methods in different external quality control sample groups (I, II, III). Success was compared between sample groups.

Test method	GROUP I		GROUP II		GROUP III		Total	
	Positive, recent EBV infection	Success %	Negative, no EBV antibodies	Success %	Negative, old EBV immunity	Success %	Total	Success %
Latex agglutination test	208/209	99.5	105/106	99.1	189/189	100	502/504	99.6
Immunochromatographic assay	6 878/6 903	99.6	4203/4222	99.5	6774/6834	99.1	17 855/17 959	99.4
Immunofiltration test^a^	146/146	100	126/128	98.4	126/148	86.3	398/422	94.3
All test methods	7232/7258	99.6	4434/4456	99.5	7089/7171	98.9	18 755/18 885	99.3
*P*-value	.729		.106		<.001^b^			

a The test kit only had the manufacturer’s validation for serum samples.

b Between Groups I and II.

The success rate between large and small testing sites was compared within the same test method (latex, immunochromatographic and immunofiltration methods). Statistically significant differences were not observed (data not shown).

The more detailed analyses showed that 23 (17.7%) of all 130 false results were obtained from one negative, old EBV immunity EQC sample, namely sample 6/2010. For this sample, the success rate was 89.8% (Table 3). The problem appeared with one test kit in particular, Clearview, which gave 150 true negative test results and, thus, 22 (16.9%) false positive results of the total of 172 reported results (Table 3).

**Table 3.** The correct results from the negative Epstein-Barr virus infectious mononucleosis external quality control sample representing old EBV immunity (Sample 6/2010).

Test methodTest kit (manufacturer)	Reported results	
	True/all	Success %
Latex agglutination tests for heterophile antibodies	8/8	100
Monogen (Biokit)	6/6	100
Monospot (Meridian)	2/2	100
Immunochromatographic assays for heterophile antibodies	194/217	89.4
Clearview (Unipath/Alere)	150/172	87.2
InstAlert (Innovacon)	41/42	97.6
Diaquick Mononucleosis Cassette (Dialab)	3/3	100
All test methods	202/225	89.8

## Discussion

EBV IM EQA testing was selected for this study because serological POC diagnostics to detect EBV IM is a common and well-established practice in different types of health care settings and it is done in different sized laboratories and testing sites. Especially the performance of the EBV IM heterophile antibody POC test methods was interesting to evaluate. These methods are one of the most common clinical microbiological examinations and according to THL’s register about 25 000 patient samples are tested per year for EBV IM in Finland.

In this study, data of 18 885 EQC results from 95 EBV IM EQC samples were analysed. In Finland, all testing of communicable diseases, including POC tests can only be done in testing sites that are approved for this purpose. Approval requires mandatory participation in the EQA rounds for each test type that a testing site offers. The results are from 273 Finnish testing sites which took part in Labquality Ltd.’s EQA rounds during 2010–2017.

The overall success shown by data on results of EQC samples was good, with 99.3% correctly reported EQC results. In all, 3 different test methods and 17 different test kits were used. The success rates between the different test methods varied from 94.3% to 99.6%. In addition, there were variations in success rates between the test kits. The lowest success rates were associated with negative samples and the QuickVue (82.0%) and the RDT EBV IgM Assay (91.3%) kits; the former is an immunochromatographic method for heterophile antibodies, the latter an immunofiltration method for IgM antibodies. The data showed no clear trend in quality development in EBV IM EQC test results.

Laboratory diagnosis of acute mononucleosis has been based on the detection of heterophile antibodies that are directed against the antigens found in sheep, horse and bovine erythrocytes, which are exploited in many test kits.^4,13,14^ Heterophile antibodies are usually demonstrable from the first week of the illness and decline to low levels by 3 months.^15^ There are cases, especially in children less than 4 years old, where heterophile antibodies may stay negative throughout the whole illness.^16,17^ In addition, heterophile antibody tests may be positive in other viral infections, like autoimmune diseases and haematological malignancies. Even though various more specific immunological methods for identifying EBV-related diseases have been developed, heterophile antibody test methods are still widely used to test EBV IM. This is partly because they are cheap, rapid and easy to use.

According to the manufacturers, all the test kits used in EQA rounds were suitable for POC testing. All the immunochromatographic and latex agglutination test methods used in this study were targeted to heterophile antibodies and were suitable for plasma samples. According to the manufacturers kit insert the immunofiltration RDT EBV IgM Assay has only been validated by its manufacturer (Bio-Rad) for serum samples, though it was used to analyse EQC samples made from liquid human plasma. However, while this kit succeeded in having 100% success with positive samples, it only had 91.3% success with negative samples.

Of the 17 POC test kits that were in use for EBV IM, the immunochromatographic methods were the most common: 12 test kits from 12 manufacturers. The test kit that was originally used in a testing site was often changed during the study period due to competitive tendering, reducing material costs or seeking a test method that produces better results in EQA rounds. Four test kits out of 17 were in use over the whole 8-year study period. These test kits were Monogen, Monospot, Clearview and InstAlert. Some kits were only in use for a short period of time. QuickVue (Quidel) was used in several laboratories during 2016 and 2017, and the Mono Rapid Test Cassette (Hangzhou Alltest biotech) was only used in one EQA round.

Test kit manufacturers sometimes improved their tests by making them more easy to interpret by the user. When choosing the test kit, laboratories often consider performance, ease of use, turnaround time and costs. Due to this, the best performing test kit is not always the most commonly used one. All the test kits had the CE marking for in vitro professional use. In Finland, as an EU member, POC tests are regulated by the European directive on CE marking, which simply certifies that a product meets basic EU health, safety and environmental standards.^18^ Even in this situation, proper validation of the conditions in which the test is intended to be used and regular participation in EQA rounds are important in order to ensure the good quality of the results of patient specimens.

For the further analyses, the EQC samples were divided into 3 groups according to the clinical interpretation obtained from pretesting, which consisted of an investigation of heterophile antibodies, EBV-IgM, EBV nuclear antigen EBNA-IgG, EBV-IgG antibodies and the avidity of IgG antibodies. The presence of the IgM antibody in the EBV viral capsid antigen, EBV-VCA, is the most important serologic finding in acute primary EBV infection. The IgG antibody to the EBV nuclear antigen, EBNA, is usually absent in the acute phase of EBV infection but, once formed, persists for the rest of a person’s life. The presence of IgG antibodies in the EBV viral capsid antigen EBV-VCA indicates that an EBV infection has occurred either recently or further into the past.^19,20^ The IgG avidity is low at the beginning of the infection but increases when the immune response matures.^21^ The results of the EQC samples that represented acute EBV infection or were negative for EBV antibodies were the easiest to interpret correctly. The samples that represented old EBV immunity were the ones that gave most of the false positive results.

According to Green,^22^ more than 70% of laboratory-related errors take place before performing the actual test. This includes mixing up samples and choosing the wrong specimen type for the test. Both of these were seen in this study. There were 4 potential mix-ups that yielded 4 false positive results and 4 false negative results with the immunochromatographic test methods. The RDT EBV IgM Assay (Bio-Rad) test kit was used to analyse EBV IM EQC plasma samples although the manufacturer had only validated its use for serum samples. Sometimes laboratories may validate more sample types than the manufacturer has initially done. There is no clear answer to how a different sample matrix affects the results. In this study, however, the testing sites that used the RDT EBV IgM Assay (immunofiltration method) reported almost 9% false positive results for negative EBV samples and even almost 14% false positive results for the specimens that represented old EBV immunity compared to latex (0% false) and immunochromatographic test methods (1% false). In all, it was challenging to correctly test the samples representing old immunity and the results were statistically significantly more often wrong compared to samples representing clearly positive or negative samples.

All personnel conducting various laboratory and POC tests should be appropriately trained and the results properly monitored. Serological POC tests are designed to be easy to use and many are visually read. Visual reading and interpretation of the test result requires skills and experience. In the POC test, a result is often qualitative, thus making the interpretation of the test result even more critical. The evaluation of the results of one of the most common clinical microbiology POC test, GAS (Group A Streptococcus) antigen detection, has shown that laboratory personnel are better at producing the right answer with POC tests than nursing staff.^23,24^ However, there are also some recorded mononucleosis pseudo epidemics in the literature due to tests that have been falsely interpreted as positive by laboratory personnel.^25,26^

From the total of 130 reported false EQC results 46 were from EQA rounds that yielded one or two reported false results per round. The randomness of these few scattered false results might indicate that there were some troubles in conducting the test or interpreting the result at that particular time. In the case of a real patient sample, it is important to remember that, if needed, there are several other, more specific laboratory tests, such as tests for EBV-specific antibodies, to confirm suspicious heterophile antibody test results. The possibility of false negative results in the case of young children and the possibility of false positive results if the clinical and haematological findings do not support the diagnosis should be kept in mind when interpreting the test results.^27^ One should also continuously monitor the level of positive and negative results per tested sample for changes (an unexpected increase or decrease) in order to investigate the cause of any such phenomena.

In EQC Sample 6/2010, only 89.8% of the results were correct, suggesting that there might be some problems with this sample or the test kits used by the testing sites. It turned out that almost 13% of the wrong results (false positives) were due to the Clearview test kit. The problem was seen only in Finnish testing sites, although foreign testing sites also investigated this sample (information from Labquality). This led to the conclusion that there might have been a problem in a single reagent lot. The lot-to-lot analytical variation of immunoassays has been noted and accepted before,^28,29^ and it has been stated that it would be useful to mark the lot information in EQA rounds.^30^

In this study, the testing sites were grouped according to the number of EBV IM investigations for patient specimens they conducted per year. Based on the collected data, the EQC results are consistent regardless of the site of testing. Having a large testing site with a wide range of different patient specimens and test methods in use, and thus, experienced personnel, did not seem to affect the EBV IM test results as much as the used test kit.

EQC results can be seen to reflect the everyday work in a clinical laboratory or testing site. In this study, there were some differences in the performance of the used test methods. Also, a sample’s clinical status affected the performance. Within those differences was the user’s impact on the test reliability. Internal controls and EQA rounds help a testing site to see whether tests work as they are meant to work in the conditions in which they are being used. The quality of laboratory diagnostics is dependent on the sample gathering, laboratory process and interpretation of the reported results in the clinic. When new test methods and test kits arrive, it might take some time to get to know their behaviour in different situations. Differences between test methods should also be known by the laboratory and the clinician, and the clinician should use appropriate clinical criteria when ordering laboratory tests, as this can have an effect on the reliability of the results. Therefore, good collaboration between the laboratory and clinic is one of the key elements to ensure good management, patient care and patient safety.

## References

[1] Plebani M. Clinical laboratories: production industry or medical services? Clin Chem Lab Med. 2015;53:995–1004. 25405721 10.1515/cclm-2014-1007

[2] Dunmire SK Verghese PS Balfour HH. Primary Epstein-Barr virus infection. J Clin Virol [Internet]. 2018;102:84–92. 29525635 10.1016/j.jcv.2018.03.001

[3] Svahn A Berggren J Parke A Storsaeter J Thorstensson R Linde A. Changes in seroprevalence to four herpesviruses over 30 years in Swedish children aged 9-12 years. J Clin Virol. 2006;37:118–123. 16971177 10.1016/j.jcv.2006.07.012

[4] Aronson MD Komaroff AL Pass TM Ervin CT Branch WT. Heterophil antibody in adults with sore throat. Frequency and clinical presentation. Ann Intern Med. 1982;96:505–508. 6895981 10.7326/0003-4819-96-4-505

[5] Linderholm M Boman J Juto P Linde A. Comparative evaluation of nine kits for rapid diagnosis of infectious mononucleosis and Epstein-Barr virus-specific serology. J Clin Microbiol. 1994;32:259–261. 8126196 10.1128/jcm.32.1.259-261.1994 PMC263013

[6] Elgh F Linderholm M. Evaluation of six commercially available kits using purified heterophile antigen for the rapid diagnosis of infectious mononucleosis compared with Epstein-Barr virus-specific serology. Clin Diagn Virol. 1996;7:17–21. 9077426 10.1016/s0928-0197(96)00245-0

[7] Svahn A Magnusson M Jägdahl L Schloss L Kahlmeter G Linde A. Evaluation of three commercial enzyme-linked immunosorbent assays and two latex agglutination assays for diagnosis of primary Epstein-Barr virus infection. J Clin Microbiol. 1997;35:2728–2732. 9350722 10.1128/jcm.35.11.2728-2732.1997 PMC230050

[8] Bruu A-L Hjetland R Holter E, et al Evaluation of 12 commercial tests for detection of Epstein-Barr virus-specific and heterophile antibodies. Clin Diagn Lab Immunol. 2000;7:451–456. 10799460 10.1128/cdli.7.3.451-456.2000 PMC95893

[9] FINLEX ®. Finnish Communicable Disease Act 583/1986, changed 935/2003, §10 [Internet]. 1986 Accessed February 5, 2020 http://www.finlex.fi/en/laki/kaannokset/1986/19860583

[10] Anja Siitonen PL Microbiology laboratory licenses are about to expire. (in Finnish). Moodi. 1996;6:252–255.

[11] ISO 17043:2010. General requirements for proficiency testing. 2010 https://17025store.com/iso-17025-standards/iso-iec-170432010-proficiency-testing/

[12] Haigh J Cox DR Snell EJ. Analysis of binary data. Math Gaz. 1990;74:91.

[13] Davidsohn I Walker PH. The nature of the heterophilic antibodies in infectious mononuclesis. Am J Clin Path. 1935;5:445–465.

[14] Paul JR Bunnell WW. The presence of heterophile antibodies in infectious mononucleosis. Clin Infect Dis. 1982;4:1062–1068. 6755615

[15] Niederman JC McCollum RW Henle G Henle W. Infectious mononucleosis. Br Med J. 1963;203:139–143. 10.1001/jama.203.3.205 4864269

[16] Topp SK Rosenfeldt V Vestergaard H Christiansen CB Von Linstow ML. Clinical characteristics and laboratory findings in Danish children hospitalized with primary Epstein-Barr virus infection. Infect Dis (Auckl). 2015;47:908–914. 10.3109/23744235.2015.1082036 26308113

[17] Sumaya CV Ench Y. Epstein-Barr virus infectious mononucleosis in children. II. Heterophil antibody and viral-specific responses. Pediatrics. 1985;75:1011–1019. 2987785

[18] Directive 98/79/EC of the European Parliament and of the Council of 27 October 1998 on in vitro diagnostic medical device. Off J Eur Communities. 1998 https://eur-lex.europa.eu/legal-content/EN/TXT/?uri=OJ:L:1998:331:TOC

[19] Nikoskelainen J Leikola J Klemola E. Igm antibodies specific for Epstein-Barr virus in infectious mononucleosis without heterophil antibodies. Br Med J. 1974;4:72–75. 4370085 10.1136/bmj.4.5936.72 PMC1612132

[20] Blake JM Edwards JMB Fletcher W McSwiggan DA Pereira MS. Measurement of heterophil antibody and antibodies to EB viral capsid antigen IgG and IgM in suspected cases of infectious mononucleosis. J Clin Pathol. 1976;29:841–847. 185241 10.1136/jcp.29.9.841 PMC476190

[21] Gray JJ Wreghitt TG. Immunoglobulin G avidity in Epstein-Barr virus infections in organ transplant recipients. Serodiagn Immunother Infect Dis [Internet]. 1989;3:389–393.

[22] Green SF. The cost of poor blood specimen quality and errors in preanalytical processes. Clin Biochem [Internet]. 2013;46:1175–1179. 23769816 10.1016/j.clinbiochem.2013.06.001

[23] Fox JW Cohen DM Marcon MJ Cotton WH Bonsu BK. Performance of rapid streptococcal antigen testing varies by personnel. J Clin Microbiol. 2006;44:3918–3922. 16971645 10.1128/JCM.01399-06 PMC1698329

[24] Nissinen A Strandén P Myllys R Takkinen J Björkman Y Leinikki P, et al Point-of-care testing of group A streptococcal antigen: performance evaluated by external quality assessment. Eur J Clin Microbiol Infect Dis. 2009;28:17–20. 18604573 10.1007/s10096-008-0580-9

[25] Armstrong CW Hackler RL Miller GB. Two pseudo-outbreaks of infectious mononucleosis. Pediatr Infect Dis. 1986;5:325–327. 3014455 10.1097/00006454-198605000-00010

[26] Herbert JT Feorino P Caldwell GG. False-positive epidemic infectious mononucleosis. Am Fam Physician. 1977;15:119–121. 835452

[27] Hess RD. Routine Epstein-Barr virus diagnostics from the laboratory perspective: still challenging after 35 years. J Clin Microbiol. 2004;42:3381–3387. 15297472 10.1128/JCM.42.8.3381-3387.2004 PMC497621

[28] Hölzel W. Analytical variation in immunoassays and its importance for medical decision making. Scand J Clin Lab Invest Suppl. 1991;205:113–119. 1947740 10.3109/00365519109104609

[29] Thompson S Chesher D. Lot-to-lot variation. Clin Biochem Rev. 2018;39:51–60. 30473592 PMC6223607

[30] Stavelin A Riksheim BO Christensen NG Sandberg S. The importance of reagent lot registration in external quality assurance/proficiency testing schemes. Clin Chem. 2016;62:708–715. 26980211 10.1373/clinchem.2015.247585

